# Optical control of levitated nanoparticles via dipole–dipole interaction

**DOI:** 10.1515/nanoph-2024-0287

**Published:** 2025-03-24

**Authors:** Sandeep Sharma, Seongi Hong, Andrey S. Moskalenko

**Affiliations:** Department of Physics, KAIST, Daejeon 34141, Republic of Korea

**Keywords:** optomechanics, levitated optomechanics, unidirectional transport, phonon lasing, mechanical squeezing, bistability

## Abstract

We propose a scheme to create and unidirectionally transport thermal squeezed states and random-phase coherent states in a system of two interacting levitated nanoparticles. In this coupled levitated system, we create a thermal squeezed state of motion in one of the nanoparticles by parametrically driving it and then transporting the state to the other nanoparticle by making use of a unidirectional transport mechanism. This mechanism is based on inducing a nonreciprocal type of coupling in the system by suitably modulating the phases of the trapping lasers and the interparticle distance between the levitated nanoparticles. A nonreciprocal coupling creates a unidirectional channel where energy flows from one nanoparticle to the other nanoparticle but not vice versa, thereby allowing for the transport of mechanical states between the nanoparticles. We also affirm this unidirectional transport mechanism by creating and efficiently transporting a random-phase coherent state in the coupled levitated system. In both instances of mechanical state transport, the final nanoparticle showed similar characteristics to the original nanoparticle, depicting a high-fidelity unidirectional transport mechanism. Further, we make use of the feedback nonlinearity and parametric driving to create simultaneous bistability in the coupled levitated system also via this unidirectional mechanism. Our results may have potential applications in tunable sensing, metrology, quantum networks, and in exploring many-body physics under a controlled environment.

## 1 Introduction

Optically levitated single nanoparticles, owing to their high tunability and low decoherence, have emerged as an ideal candidate for applications in high precision sensing [1], [2], testing fundamental limits in physics [3], [4], [5], [6], and exploring nonequilibrium physics [7]. Further, with recent developments toward trapping of multiple nanoparticles, a new avenue has been opened up, which holds potential for exploration of coupled dynamics using these levitated nanoparticle arrays [8], [9], [10], [11]. To this end, efforts have been made to study the optical binding interaction and Coulomb interaction between two levitated nanoparticles [9], [10], [11], simultaneous cooling of the mechanical motion of two levitated nanoparticles [12], [13], [14], [15], stochastic dynamics [16], synchronization [17], manipulating coupling interaction between the nanoparticles [18], entanglement dynamics [19], [20], quantum correlations between levitated nanoparticles [21], differential force sensing [22], and Hermitian and non-Hermitian physics [23], [24], [25].

In this work, we mainly focus on the coupled dynamics of optically interacting two levitated nanoparticles. Optically interacting levitated systems (OILSs) are highly tunable and hold a greater advantage over systems interacting via the Coulomb force as concerns their utilization as a platform for exploring various many-body physics. This is because the coupling strength between the levitated nanoparticles in OILSs can be efficiently controlled by modulating the intensity of trapping lasers, their phases, and the interparticle distance [10], while such a high degree of control is limited in the systems interacting via the Coulomb force [11]. Recently, a full quantum model to study the dynamics of OILSs was proposed by Rudolph et al. [26], [27]. One of the interesting results originating from the quantum description of optical interaction is that the coupled nanoparticles experience correlated quantum noises. This is an intriguing phenomenon that may help to enhance the synchronization of coupled systems [28]. Further, using this quantum model, Rudolph et al. have discussed the prospect of studying two-mode squeezing, possibility of entanglement, and unidirectional quantum transport in these OILSs. Toward unidirectional quantum transport, they mentioned that the corresponding effects may be possible by suitably modulating the trapping laser phases and the interparticle distance. However, a detailed discussion of this transport mechanism in OILSs has remained unexplored. Achieving unidirectional transport in OILSs can be an important development toward demonstration of directional amplification [29], [30] and exploring topological effects [31], [32].

Motivated by this, we study the creation of different mechanical states in two optically interacting levitated nanoparticles, as considered in Ref. [10], and explore the possibility of transporting such states from one nanoparticle to the other. We start by creating a thermal squeezed state of motion in one of the nanoparticles via a parametric drive. Next, by inducing a unidirectional coupling in the system, we find that the thermal squeezed state can be transported to the other nanoparticle with a very high fidelity. Further, to demonstrate the effectiveness of our unidirectional transport scheme, we also study the creation and transport of a random-phase coherent state in the coupled system and find a high-fidelity induced transport rate for this case as well. Finally, we utilize this unidirectionality phenomenon to generate simultaneous bistability in the studied coupled levitated system.

## 2 Theoretical model

**Figure 1:**
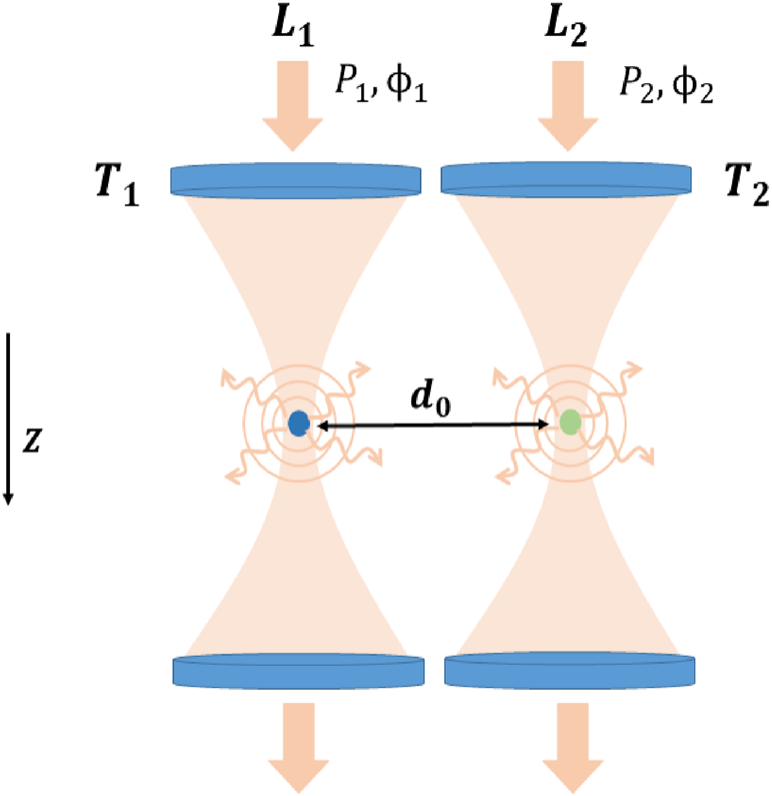
A schematic diagram of a system of two interacting levitated nanoparticles. The nanoparticles labeled as blue and green dots are trapped at an interparticle distance *d*_0_ by two optical tweezers *T*_1_ and *T*_2_. Both nanoparticles interact with each other via photons scattered from trapping lasers *L*_1_ and *L*_2_ having powers *P*_1_ and *P*_2_ and phases *ϕ*_1_ and *ϕ*_2_, respectively.

We consider two dielectric nanoparticles having mass *m* and radius *r* that are levitated in different optical trap potentials created by two distinct optical tweezers, as shown in Figure 1. Both nanoparticles interact with each other via the scattered fields from one another, giving rise to a nonreciprocal type of optical binding force between them [10]. To a good approximation, the trapping potentials can be considered harmonic [1]; hence, the two levitated nanoparticles can be seen as two interacting harmonic oscillators (HOs).

The quantum dynamics of these interacting HOs can be captured by the following master equation [26]:

(1) ρ˙=∑j=12−iωjbj†bj+Sj4Qzj2,ρ−∑j=12iγgj2[Qzj,{Pzj,ρ}]+∑j=12DtjD[Qzj]ρ+ig12[Qz1Qz2,ρ]+∑j,j′=1j≠j′2Djj′QzjρQzj′−12QzjQzj′,ρ,

where *ρ* represents the two-particle density matrix for the coupled levitated system [see Eq. (A-1) for full master equation]. The dimensionless position and momentum operators for the nanoparticles are designated by $Q_{z_{j}}=\left(b_{j}^{†}+b_{j}\right)$ and $P_{z_{j}}=i\left(b_{j}^{†}-b_{j}\right)$ , respectively, with $b_{j}^{†}$ and *b*_*j*_ denoting the phonon creation and annihilation operators of the mechanical mode of the nanoparticles. Further, both $b_{j}^{†}$ and *b*_*j*_ obey the bosonic commutation relation $\left[b_{j},b_{j^{\prime }}^{†}\right]=\delta_{jj^{\prime }}$ . The Lindblad superoperator D[O] acts on *ρ* as follows: $D\left[O\right]\rho =O\rho O^{†}-\frac{1}{2}O^{†}O\rho -\frac{1}{2}\rho O^{†}O$ .

The first term on the right-hand side (rhs) of Eq. (1) consists of two parts. The first part corresponds to the harmonic motion of both nanoparticles with frequencies *ω*_1_ and *ω*_2_, respectively. The second part represents the effect of optical binding force on the motion of the nanoparticle with strengths *S*_1_ and *S*_2_, respectively. The second term on the rhs of Eq. (1) reflects the damping of nanoparticle motion with a rate *γ*_*gj*_ due to the surrounding gas. The third term represents the effect of photon scattering (*A*_*tj*_) and gas scattering (*D*_*pj*_) on the motion of nanoparticles with the total rate *D*_*tj*_ = *A*_*tj*_ + *D*_*pj*_ [33]. The fourth term is responsible for reciprocal coupling between the nanoparticles with strength *g*_1_. The fifth term represents the correlation between scattering noises experienced by the nanoparticles (real part of *D*_*jj*′_) and the nonreciprocal coupling (imaginary part of *D*_*jj*′_) between them. The strengths of the optical binding force, *S*_1_ and *S*_2_, depend on the interparticle distance *d*_0_ and relative phase difference between trapping lasers Δ*ϕ* = *ϕ*_1_ − *ϕ*_2_ and are given by *S*_1_ = *g*_1_ + *g*_2_ and *S*_2_ = *g*_1_ − *g*_2_, with *g*_1_ = *g* cos(*kd*_0_)cos(Δ*ϕ*)/*kd*_0_ and *g*_2_ = *g* sin(*kd*_0_)sin(Δ*ϕ*)/*kd*_0_. Here, *g* is the modulating constant given by $g=\alpha^{2}k^{3}{\left(k-1/z_{R}\right)}^{2}\sqrt{P_{1}P_{2}}/\left(2c\omega_{b}^{2}\pi^{2}\epsilon_{0}^{2}\right)$ , where *α* is the polarizability of nanoparticles, *k* is the wave vector of trapping lasers, *P*_1_(*P*_2_) is the power of the trapping laser beam 1 (2), *ω*_*b*_ is the beam waist, *c* is the speed of light, and *ϵ*_0_ is the vacuum permittivity [10]. Further, the complex quantity *D*_12_(*D*_21_) is expressed as $D_{12}=D_{21}^{*}=J_{s}+ig_{2}/2$ , where “∗” denotes complex conjugate, *J*_*s*_ = *g* sin(*kd*_0_)cos(Δ*ϕ*)/*kd*_0_ represents the strength of noise correlation, and *g*_2_ is the strength of nonreciprocal coupling.

At first, we try to gain a basic understanding of the dynamics of a system of two interacting levitated nanoparticles in the absence of any kind of external drive or feedback. For this, we analyze the stochastic equations of motion (EOMs) for the coupled levitated system, which are obtained from Eqs. (B-5) and (B-6), and are described as

(2) $$\begin{array}{ll} {\ddot{Q}}_{z_{1}} & =-\omega_{1}^{2}Q_{z_{1}}-2\gamma_{g1}{\dot{Q}}_{z_{1}}-\omega_{1}S_{1}Q_{z_{1}}\\ & \;+\omega_{1}S_{1}Q_{z_{2}}+\omega_{1}F_{1}, \end{array}$$

(3) $$\begin{array}{ll} {\ddot{Q}}_{z_{2}} & =-\omega_{2}^{2}Q_{z_{2}}-2\gamma_{g2}{\dot{Q}}_{z_{2}}-\omega_{2}S_{2}Q_{z_{2}}\\ & \;+\omega_{2}S_{2}Q_{z_{1}}+\omega_{2}F_{2}, \end{array}$$

where $F_{1}$ and $F_{2}$ are the Langevin forces acting on nanoparticle 1 and 2, respectively. Further, it should be noted that in the above equations, the stochastic noises determined by the Langevin forces are correlated. A detailed discussion on this can be found in Ref. [26]. It is evident from the expressions for *S*_1_ and *S*_2_ that for *kd*_0_ = 2*nπ* + *π*/4 and Δ*ϕ* = 2*nπ* + *π*/4, with *n* being a non-negative integer, Eq. (3) becomes independent of $Q_{z_{1}}$ , while Eq. (2) remains dependent on $Q_{z_{2}}$ . This implies that for the aforementioned condition, a unidirectional coupling is induced in the system, where there is an energy flow from particle 2 to particle 1 but not vice versa [34]. In the next section, we will utilize this unidirectionality phenomenon and explore the possibility of transporting different mechanical states from one particle to another.

## 3 State creation and transport

In this section, we demonstrate the creation of a squeezed thermal state and a random-phase coherent state in the mechanical mode of nanoparticle 2 as well as their transport to nanoparticle 1 via a unidirectional coupling with high fidelity. Additionally, we also show simultaneous bistable dynamics in both levitated nanoparticles due to this coupling.

### 3.1 Squeezed state

To create a squeezed state of motion for nanoparticle 2, we parametrically drive it with a force having strength $f\omega_{2}^{2}$ and tuned at twice the oscillation frequency of the nanoparticle [35]. Under the action of this force along with the condition *S*_2_ = 0, the EOMs for the levitated nanoparticles can be written as

(4) $${\ddot{Q}}_{z_{1}}=-\omega_{1}^{2}Q_{z_{1}}-\gamma_{g1}{\dot{Q}}_{z_{1}}-2\omega_{1}sQ_{z_{1}}+2\omega_{1}sQ_{z_{2}}+\omega_{1}F_{1},$$

(5) $${\ddot{Q}}_{z_{2}}=-\omega_{2}^{2}Q_{z_{2}}-\gamma_{g2}{\dot{Q}}_{z_{2}}-f\omega_{2}^{2}\sin\left(2\omega_{2}t\right)Q_{z_{1}}+\omega_{2}F_{2},$$

where *s* = *g*/*kd*_0_ [see Eqs. (B-5) and (B-6) in Appendix B]. In the above equations, we have considered that the linear feedback heating (*γ*_*a*_), nonlinear feedback cooling (*γ*_*f*_), and their backactions (Γ_*a*_) and (Γ_*f*_), respectively, are absent. For simplicity, we then assume that both nanoparticles have the same frequency *ω*_1_ = *ω*_2_ = *ω*_0_ and are subjected to the same damping *γ*_*g*1_ = *γ*_*g*2_ = *γ*_*g*_. We also assume equal scattering rates *A*_*t*1_ = *A*_*t*2_ = *A*_*t*_ for both nanoparticles. This situation can be easily created in experiments by controlling the trapping laser intensity and the gas pressure [10], [33]. To visualize the creation and transport of thermal squeezed states, we study the phase-space dynamics of the coupled levitated system by making the following ansatz for the solution of Eqs. (4) and (5):

(6) $$Q_{z_{j}}=Q_{j}\;\cos\left(\omega_{0}t\right)+P_{j}\;\sin\left(\omega_{0}t\right),\;\;\;\;j=1,2.$$

**Figure 2:**
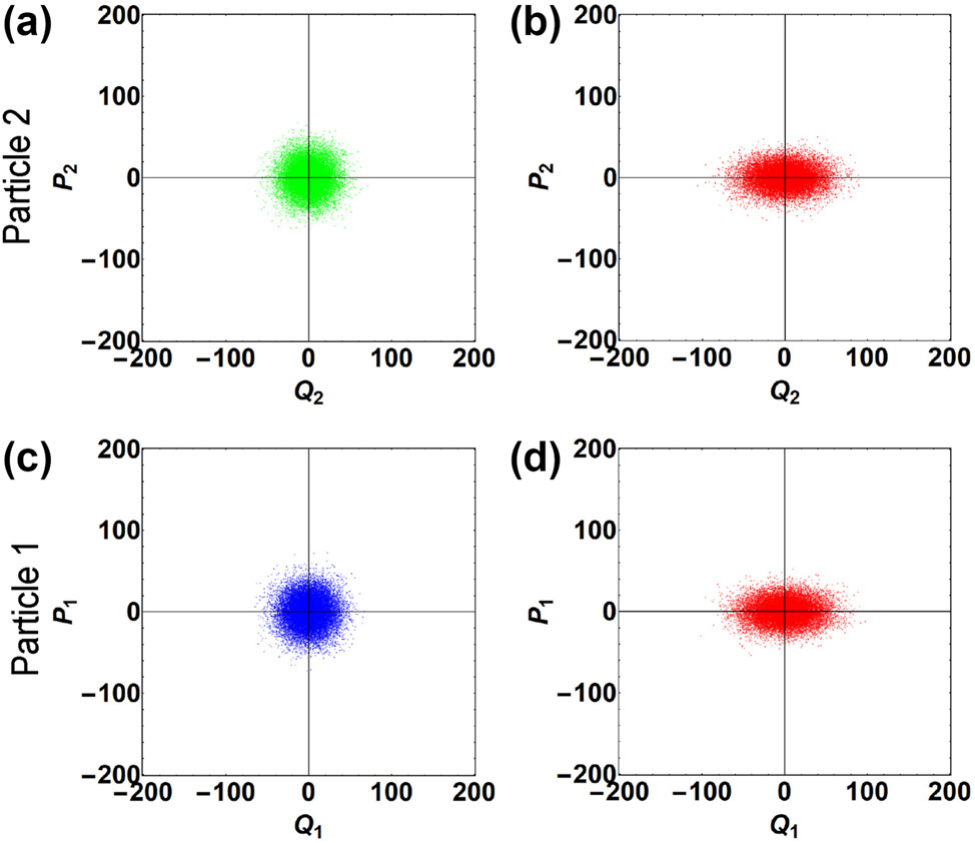
Projected motion of both nanoparticles in phase space. Panel (a) [(b)] shows the initial [final] state of motion of nanoparticle 2. Panel (c) [(d)] shows the state of nanoparticle 1 before [after] a unidirectional coupling. Parameters: *ω*_0_ = 127 kHz, *A*_*t*_ = 1 kHz, *γ*_*g*_ = 1 Hz, *r* = 0.8, *γ*_*a*_ = 0 Hz, *γ*_*f*_ = 0 Hz, Γ_*a*_ = 0 Hz, Γ_*f*_ = 0 Hz, *s* = 50 Hz, and *J*_*s*_ = 25 Hz, corresponding to experimental values from Refs. [10], [33].

Here, *Q*_*j*_ and *P*_*j*_ are slowly varying quadrature components of the motion of the nanoparticles [36], [37]. Next, by utilizing Eqs. (6) in (4) and (5), we can write the EOMs for the quadrature components of both nanoparticles as

(7) $${\dot{Q}}_{1}=-\gamma_{g}Q_{1}+sP_{1}-sP_{2}-\frac{F_{s1}}{2},$$

(8) $${\dot{P}}_{1}=-\gamma_{g}P_{1}-sQ_{1}+sQ_{2}+\frac{F_{c1}}{2},$$

(9) $${\dot{Q}}_{2}=-\left(\gamma_{g}-r\gamma_{g}\right)Q_{2}-\frac{F_{s2}}{2},$$

(10) $${\dot{P}}_{2}=-\left(\gamma_{g}+r\gamma_{g}\right)P_{2}+\frac{F_{c2}}{2},$$

where *r* = *fω*_0_/*γ*_*g*_ is the squeezing strength, and $F_{jc}$ and $F_{js}$ are slowly varying cosine and sine components of the Langevin forces, respectively [see Eqs. (B-9)–(B-12) in Appendix B]. To numerically solve Eqs. (7)–(10), we follow the approach as in Refs. [37], [38], [39] and present the solution obtained for the long-time interaction limit in the form of phase-space plots as shown in Figure 2. In Figure 2, panels (a) and (b) show the phase-space distribution of the motion of nanoparticle 2 before and after the parametric driving, respectively. Figure 2(a) shows a circularly symmetric distribution indicating that nanoparticle 2 is in a thermal state, which is expected as initially the nanoparticle motion is solely driven by thermal Langevin forces [37]. From Figure 2(b), it is evident that when a parametric drive is applied, the thermal fluctuations along one of the quadrature components are amplified, while along the other component, they are deamplified, resulting in a squeezed distribution. This reflects the creation of a thermal squeezed state of motion of nanoparticle 2. To quantify the squeezing induced in the system, we now evaluate the variances of the quadrature components of the motion of nanoparticle 2. Under the condition *A*_*t*_ ≫ *J*_*s*_, they are given by

(11) $$\sigma_{Q_{2}}^{2}=\frac{1}{\pi }\overset{\infty }{\underset{-\infty }{\int }}〈|Q_{2}\left(\omega \right){|}^{2}〉d\omega =\frac{A_{t}}{4\gamma_{g}\left(1-r\right)},$$

(12) $$\sigma_{P_{2}}^{2}=\frac{1}{\pi }\overset{\infty }{\underset{-\infty }{\int }}〈|P_{2}\left(\omega \right){|}^{2}〉d\omega =\frac{A_{t}}{4\gamma_{g}\left(1+r\right)}.$$

Here, *Q*_2_(*ω*) and *P*_2_(*ω*) are derived by solving Eqs. (9) and (10) in the frequency domain [36], [37], [40]. Since, only *J*_*s*_ is dependent on *d*_0_, the condition *A*_*t*_ ≫ *J*_*s*_ can be achieved by increasing the interparticle distance *d*_0_, while keeping the condition *kd*_0_ = 2*nπ* + *π*/4 intact for unidirectionality. From Eqs. (11) and (12), it is apparent that when the squeezing strength *r* vanishes, the variances of both quadrature components become equal. This indicates that the phase-space distribution has to be symmetric, which is in accordance with the result shown in Figure 2(a). For *r* ≠ 0, the variance of quadrature *Q*_2_ is greater than that of the *P*_2_, corresponding to a squeezed distribution in the phase space, as shown in Figure 2(b).

Next, we initiate a unidirectional coupling and study the phase-space dynamics of the motion of nanoparticle 1. It is clear from Figure 2 that in the presence of a unidirectional coupling, the state of nanoparticle 1 evolves from a thermal state, as shown in Figure 2(c), to a squeezed state revealed in Figure 2(d). Further, following a similar approach as mentioned above, we quantify squeezing in the motion of nanoparticle 1 by deriving the variances of its quadrature components. Assuming that both nanoparticles interact for a long time, eventually reaching a steady state, under the condition, *A*_*t*_ ≫ *J*_*s*_ and *s* > *γ*_*g*_, we write the variances as

(13) $$\begin{array}{ll} \sigma_{Q_{1}}^{2} & =\frac{1}{\pi }\overset{\infty }{\underset{-\infty }{\int }}\left\langle |Q_{1}\left(\omega \right){|}^{2}\right\rangle d\omega \\ & \approx \frac{A_{t}}{4\gamma_{g}\left(1-r^{2}\right)s^{2}}\left[\gamma_{g}^{2}\left(1-r\right)+s^{2}\left(1+r\right)\right]\\ & \approx \frac{A_{t}}{4\gamma_{g}\left(1-r\right)}\;\;\;\;\text{for}\;\;s\gg \gamma_{g}, \end{array}$$

(14) $$\begin{array}{ll} \sigma_{P_{1}}^{2} & =\frac{1}{\pi }\overset{\infty }{\underset{-\infty }{\int }}\left\langle |P_{1}\left(\omega \right){|}^{2}\right\rangle d\omega \\ & \approx \frac{A_{t}}{4\gamma_{g}\left(1-r^{2}\right)s^{2}}\left[\gamma_{g}^{2}\left(1+r\right)+s^{2}\left(1-r\right)\right]\\ & \approx \frac{A_{t}}{4\gamma_{g}\left(1+r\right)}\;\;\;\;\text{for}\;\;s\gg \gamma_{g}. \end{array}$$

**Figure D-6:**
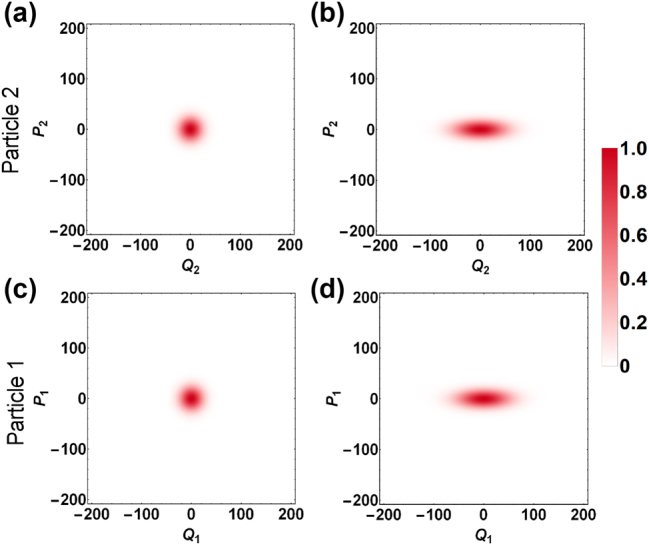
Phase-space distribution of motion of both the nanoparticles plotted using analytical expressions of P as in Eqs. (D-1) and (D-2). Panel (a) [(b)] shows the state of nanoparticle 2 before [after] parametric driving. Panel (c) [(d)] represents the state of nanoparticle 1 before [after] coupling. All parameters are the same as in Figure 2.

From the analysis of both the phase-space distribution and the variances of the quadrature components of both nanoparticles, we can confirm that a unidirectional coupling induces the transport of mechanical states from nanoparticle 2 to nanoparticle 1. Additionally, to verify the validity of our numerical results on the induced state transport, we compare them with corresponding analytical results obtained by solving the Fokker–Planck equation in the steady-state limit [see Appendix C & D]. Toward this, we found excellent agreement between the numerical and analytical results on the phase-space distribution [see Figure D-6] and the variances [see Eqs. (D-4)–(D-7)], which affirms the validity of our analysis elucidating such state transport mechanism in coupled levitated systems. Further, to determine the efficiency of this induced mechanical state transport process, we find the fidelity by using Eq. (D-8), which quantifies the degree of similarity between the transported state and the original state of the nanoparticles. In our case, by considering achievable experimental values of parameters [10], [33], we found the fidelity F=0.999 , reflecting a very highly efficient transport process.

### 3.2 Random-phase coherent state

In this section, to demonstrate that OILSs can be used for transporting other mechanical states, we also study the creation and transport of random-phase coherent states [41], [42] in this setting. Similar to the above subsection, we first create a random-phase coherent state of motion in nanoparticle 2 and then transport it to nanoparticle 1 via a unidirectional coupling. To create a random-phase coherent state of motion in nanoparticle 2, we simultaneously use linear feedback heating and nonlinear feedback cooling to control its motional dynamics. To understand the effect of both these feedbacks on the motion of nanoparticle 2, we numerically solve the following EOMs for the quadrature components of the nanoparticles [see Eqs. (B-9)–(B-12) in Appendix B]

(15) $${\dot{Q}}_{1}=-\gamma_{g}Q_{1}+sP_{1}-sP_{2}-\frac{F_{s1}}{2},$$

(16) $${\dot{P}}_{1}=-\gamma_{g}P_{1}-sQ_{1}+sQ_{2}+\frac{F_{c1}}{2},$$

(17) $${\dot{Q}}_{2}=-\left(\gamma_{g}-\gamma_{a}+6\gamma_{f}\left[Q_{2}^{2}+P_{2}^{2}\right]\right)Q_{2}-\frac{F_{s2}}{2},$$

(18) $${\dot{P}}_{2}=-\left(\gamma_{g}-\gamma_{a}+6\gamma_{f}\left[Q_{2}^{2}+P_{2}^{2}\right]\right)P_{2}+\frac{F_{c2}}{2}.$$

**Figure 3:**
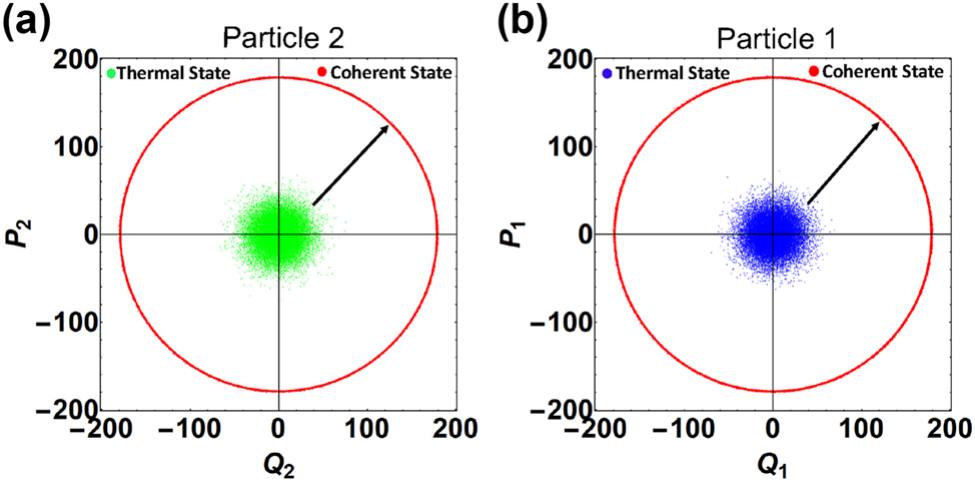
Induced transfer of a random-phase coherent state between the nanoparticles of the coupled levitated system. Panel (a) [(b)] depicts the initial thermal state and final random-phase coherent state of motion of nanoparticle 2 [1]. The arrows indicate the time evolution of the states of nanoparticles. Parameters: *γ*_*a*_ = 20 Hz, *r* = 0, *γ*_*f*_ = 10^−4^ Hz, Γ_*a*_ = 0.1 Hz, and Γ_*f*_ = 10^−6^ Hz. Other parameters are the same as in Figure 2.

The result of our numerical solution is portrayed in the phase space as shown in Figure 3. It can be seen in Figure 3(a) that, in the presence of linear feedback heating and nonlinear feedback cooling, the state of motion of nanoparticle 2 evolves from the thermal state to a random-phase coherent state. This is because the competition between gain (due to feedback heating) and loss (due to nonlinear feedback cooling) drives the motion of nanoparticle 2 toward a stable oscillation.

**Figure 4:**
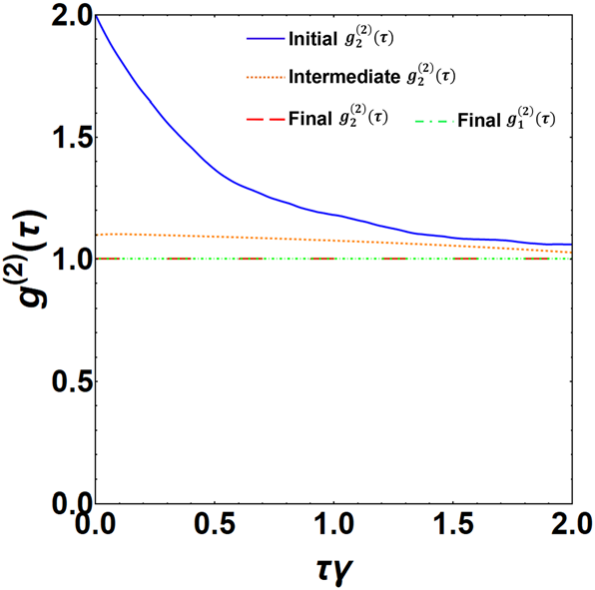
Second-order coherence $g_{2}^{\left(2\right)}\left(\tau \right)\;\left[g_{1}^{\left(2\right)}\left(\tau \right)\right]$ for nanoparticle 2 [1]. The blue solid [red long-dashed] line depicts the numerically evaluated $g_{2}^{\left(2\right)}\left(\tau \right)$ for the initial [final] state of nanoparticle 2. $g_{2}^{\left(2\right)}\left(\tau \right)$ evaluated at an intermediate time moment *τ* = 2/*γ* with an averaging time window of 4/*γ* during the evolution of nanoparticle 2 is represented by the orange dotted line. The green dotted-dashed line shows $g_{1}^{\left(2\right)}\left(\tau \right)$ for the final state of nanoparticle 1. The parameters considered here are the same as in Figure 3.

**Figure E-7:**
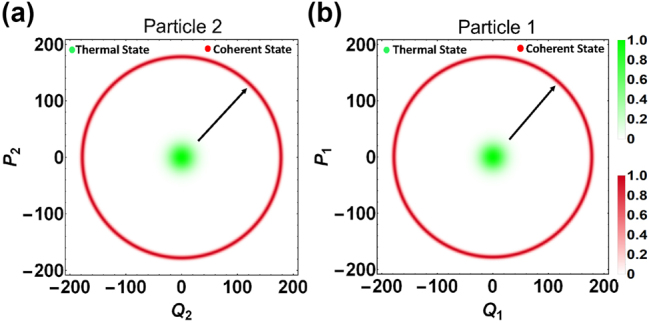
Phase-space distribution of motion of both the nanoparticles plotted using analytical expressions of P as in Eqs. (E-1) and (E-2). Panel (a) [(b)] shows the transition from the initial thermal state to a random-phase coherent state of nanoparticle 2 [1]. The arrow depicts the time evolution of the mechanical state. Parameters are the same as in Figure 3.

Next, to characterize the stable oscillation motion of nanoparticle 2, we also study the phonon population and second-order coherence. By analyzing Eqs. (17) and (18) in the steady state limit, we found that the phonon population saturates to a value $\approx \left(\gamma_{a}-\gamma_{g}\right)/6\gamma_{f}$ . We also studied the second-order coherence $g_{2}^{\left(2\right)}\left(\tau \right)$ using the approach as in Ref. [43]. The results are illustrated in Figure 4. It is clear that $g_{2}^{\left(2\right)}\left(\tau \right)$ evolves from a Lorentzian profile [for a thermal state] to a constant profile depicting nanoparticle 2 in a random-phase coherent state. Thus, the above analysis on stable oscillation dynamics, phonon saturation effect, and second-order coherence together evidence the creation of a random-phase coherent state of motion in nanoparticle 2 [41], [42]. Now, we switch on a unidirectional coupling and study the dynamics of nanoparticle 1 using Eqs. (15) and (16) and present our numerical results in Figure 3. It is evident from Figure 3(b) that the motion of nanoparticle 1 also shows stable oscillation due to a unidirectional coupling. Further, the second-order coherence $g_{1}^{\left(2\right)}\left(\tau \right)$ for the nanoparticle 1 converges to a constant profile, representing a random-phase coherent state, similar to that of nanoparticle 2, as shown in Figure 4. With this, we can affirm the tunability of the induced state transport mechanism in the studied OILSs, which is enforced by a unidirectional coupling. Then, we also verify our numerically obtained results by comparing them with analytical results gained by solving the Fokker–Planck equation [see Appendix E for details]. To this end, we also find excellent agreement between the outcomes of both approaches for the phase-space dynamics [see Figure E-7 in Appendix E] as well as for the phonon population [see Eqs. (E-3) & (E-4) in Appendix E]. Additionally, we check the efficiency of the transport process by finding the corresponding fidelity using Eq. (D-8). Considering achievable experimental values of parameters, we could reach high fidelity F=0.999 , which is similar to that demonstrated in Section 3.1 for the squeezing case.

### 3.3 Bistability

In this section, we make use of the unidirectionality phenomenon as a tool to initiate simultaneous bistable dynamics of the coupled levitated system. In this regard, we first apply nonlinear feedback cooling to nanoparticle 2 and then use parametric driving to create bistable dynamics of the motion of the nanoparticle. Specifically, we numerically solve the EOMs for the quadrature components of the nanoparticles [see Appendix B for details]:

(19) $${\dot{Q}}_{1}=-\gamma_{g1}Q_{1}+sP_{1}-sP_{2}-\frac{F_{s1}}{2},$$

(20) $${\dot{P}}_{1}=-\gamma_{g1}P_{1}-sQ_{1}+sQ_{2}+\frac{F_{c1}}{2},$$

(21) $${\dot{Q}}_{2}=-\left(\gamma_{g2}-r\gamma_{g2}+6\gamma_{f}\left[Q_{2}^{2}+P_{2}^{2}\right]\right)Q_{2}-\frac{F_{s2}}{2},$$

(22) $${\dot{P}}_{2}=-\left(\gamma_{g2}+r\gamma_{g2}+6\gamma_{f}\left[Q_{2}^{2}+P_{2}^{2}\right]\right)P_{2}+\frac{F_{c2}}{2}.$$

**Figure 5:**
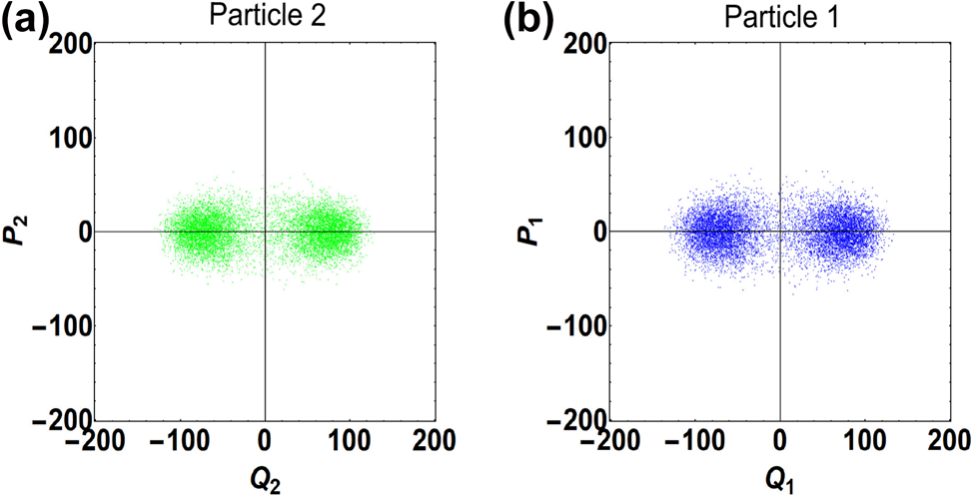
Panel (a) [(b)] shows the bistable dynamics of nanoparticle 2 [1]. Parameters: *γ*_*g*1_ = 1 Hz, *γ*_*g*2_ = 20 Hz, *r* = 0.9, *γ*_*f*_ = 2 × 10^−4^ Hz, and Γ_*f*_ = 2 × 10^−6^ Hz. Other parameters are the same as in Figure 2.

The resulting phase-space distributions are presented in Figure 5. It is apparent from Figure 5(a) that in the presence of parametric drive and nonlinear feedback cooling the motion of nanoparticle 2 shows two stable oscillations reflecting bistable dynamics. This bistability is caused by the formation of a double-well trapping potential, which can be controlled by manipulating the strength of the parametric drive as well as the nonlinear feedback cooling rate [44]. Next, by activating a unidirectional coupling channel, we observe that nanoparticle 1 also exhibits bistable dynamics, as shown in Figure 5(b).

## 4 Conclusions

In conclusion, we have explored the coupled dynamics of a system of two interacting levitated nanoparticles. We performed numerical simulations on its phase-space dynamics and showed that a unidirectional coupling enables the transport of different mechanical states from one nanoparticle to the other nanoparticle. We also made analytical calculations to illuminate the underlying transport mechanism and found it to be in excellent agreement with our results from numerical simulations. Further, from both illustrated cases of mechanical state transport, we found that the nanoparticle to which the state was transported showed similar characteristics to that of the original nanoparticle. To estimate the degree of similarity between the transported state and the original state, we determined the transport fidelity and demonstrated that its value can be very high. Finally, we also used this unidirectional mechanism to induce simultaneous bistability in the studied system. We expect that our results may have potential applications for sensing [43], [45] and may also motivate studies on synchronization of optomechanical arrays [46]. Additionally, our findings on long-lived simultaneous coherent oscillation can be extended to many-body systems wherein it may be interesting to study coherent quantum thermodynamics [47], [48] and quantum metrology [49]. Further, results on simultaneous bistable dynamics in coupled systems may motivate future explorations of many interesting nonequilibrium many-body dynamics such as quantum critical phenomena and phase transitions [50], [51].

## Appendix A: Master equation

Here, we present the full master equation, which describes the complete dynamics of the system of two interacting levitated nanoparticles [26], [33]. It reads

(A-1)
ρ˙=∑j=12−iωjbj†bj+Sj4Qzj2,ρ−iγgj2[Qzj,{Pzj,ρ}]+∑j=12DtjD[Qzj]ρ+ig12[Qz1Qz2,ρ]+∑j,j′=1j≠j′2Djj′QzjρQzj′−12QzjQzj′,ρ−ifω24sin(2ω2t)Qz22,ρ+iγa2[Qz2,{Pz2,ρ}]+2ΓaD[Qz2]ρ−iγfQz23,{Pz2,ρ}+2ΓfDQz23ρ.

The definition and effect up to the fifth term on the right-hand side (rhs) of the above equation have been explained in Section 2. The sixth term on the rhs is the parametric drive term and is responsible for generating squeezing in the system. The seventh and eighth terms represent linear feedback heating of the system with rate *γ*_*a*_ and its backaction with rate Γ_*a*_. The last two terms represent nonlinear feedback cooling of the system and its backaction with rates *γ*_*f*_ and Γ_*f*_, respectively. The backactions are expressed as Γ_*a*_ = *G*_*a*_ *γ*_*a*_ and Γ_*f*_ = *G*_*f*_ *γ*_*f*_, where *G*_*a*_ and *G*_*f*_ are the corresponding dimensionless feedback gains. The expression for the backaction terms can be derived by first detecting individual motions of nanoparticle [12], [22] and then making a homodyne measurement followed by applying the quantum theory of optical feedback for this homodyne detection [33], [52], [53]. Here, both linear feedback heating and nonlinear feedback cooling are responsible for creating a random-phase coherent state in the system. Further, in the absence of feedback heating, the parametric drive and nonlinear feedback terms give rise to bistability in the system.

## Appendix B: Langevin equation

To study the dynamics of the system, we use Eq. (A-1) to write the Langevin equations of motion for the system as presented in Refs. [54], [55]. Alternatively, they can be derived going a way via the Fokker–Planck equation as in Ref. [56]. In the limit of high phonon number [57], [58], the equations of motion can then be expressed as

(B-1)
$${\dot{Q}}_{z_{1}}=\omega_{1}P_{z_{1}},$$

(B-2)
$${\dot{P}}_{z_{1}}=-\omega_{1}Q_{z_{1}}-2\gamma_{g1}P_{z_{1}}-S_{1}Q_{z_{1}}+S_{1}Q_{z_{2}}+F_{1},$$

(B-3)
$${\dot{Q}}_{z_{2}}=\omega_{2}P_{z_{2}},$$

(B-4)
$$\begin{array}{ll} {\dot{P}}_{z_{2}} & =-\omega_{2}Q_{z_{2}}-2\left(\gamma_{g2}-\gamma_{a}+6\gamma_{f}Q_{z_{2}}^{2}\right)P_{z_{2}}\\ & \;-f\omega_{2}\sin\left(2\omega_{2}t\right)Q_{z_{2}}-S_{2}Q_{z_{2}}+S_{2}Q_{z_{1}}+F_{2}, \end{array}$$

where the Langevin forces $F_{1}$ and $F_{2}$ act on nanoparticle 1 and nanoparticle 2, respectively, and are represented as $F_{1}=\sqrt{2K_{B}T\gamma_{g1}/\hbar \omega_{1}}\;\xi_{T1}+\sqrt{D_{t1}}\;\xi_{S1}+\sqrt{J_{s}}\;\xi_{\mathrm{NC1}}$ and $F_{2}=\sqrt{2K_{B}T\gamma_{g2}/\hbar \omega_{2}}\;\xi_{T2}+\sqrt{D_{t2}}\;\xi_{S2}+\sqrt{J_{s}}\;\xi_{\mathrm{NC2}}+\sqrt{2\Gamma_{a}}\;\xi_{Ba}+12Q_{z_{2}}^{2}\sqrt{\frac{\Gamma_{f}^{2}}{\gamma_{f}}}\;\xi_{Bc}$ . Further, *ξ*_*Tj*_ and *ξ*_*Sj*_ are stochastic noises corresponding to the effects from environment and scattering, respectively. *ξ*_*NC1*_ and *ξ*_*NC2*_ represent correlated noise terms related to scattered photons from both nanoparticles. The noises from the feedback heating and nonlinear cooling are captured by *ξ*_*Ba*_ and *ξ*_*Bc*_, respectively. The noise terms possess the following correlation properties: ⟨*ξ*_*NCj*_(*t*)*ξ*_*NCj*′_(*t*′)⟩ = *δ*(*t* − *t*′), ⟨*ξ*_*ν*_(*t*)*ξ*_*ν*_(*t*′)⟩ = *δ*(*t* − *t*′), where *ν* = *Tj*, *Sj*, *Ba*, *Bc*.

We combine the four above ordinary differential equations of the first order into two equations of the second order,

(B-5)
$$\begin{array}{ll} {\ddot{Q}}_{z_{1}} & =-\omega_{1}^{2}Q_{z_{1}}-2\gamma_{g1}{\dot{Q}}_{z_{1}}-\omega_{1}S_{1}Q_{z_{1}}\\ & \;+\omega_{1}S_{1}Q_{z_{2}}+\omega_{1}F_{1}, \end{array}$$

(B-6)
$$\begin{array}{ll} {\ddot{Q}}_{z_{2}} & =-\omega_{2}^{2}Q_{z_{2}}-2\left(\gamma_{g2}-\gamma_{a}+6\gamma_{f}Q_{z_{2}}^{2}\right){\dot{Q}}_{z_{2}}\\ & \;-f\omega_{2}^{2}\sin\left(2\omega_{2}t\right)Q_{z_{2}}-\omega_{2}S_{2}Q_{z_{2}}\\ & \;+\omega_{2}S_{2}Q_{z_{1}}+\omega_{2}F_{2}. \end{array}$$

As discussed in Section 2, we consider *S*_2_ = 0, which is a prerequisite condition for a unidirectional state transport. By assuming that both nanoparticles have the same oscillation frequency *ω*_0_ and the same damping *γ*_*g*_, we can write the solution of Eqs. (B-5) and (B-6) as

(B-7)
$$Q_{z_{j}}=Q_{j}\;\cos\left(\omega_{0}t\right)+P_{j}\;\sin\left(\omega_{0}t\right),\;\;\;\;j=1,2.$$

Here, *Q*_*j*_ and *P*_*j*_ are slowly varying quadrature components of the motion of nanoparticles [37]. Further, as the nanoparticles respond at around *ω*_0_, the Langevin forces can also be written into the following form

(B-8)
$$F_{j}=F_{jc}\;\cos\left(\omega_{0}t\right)+F_{js}\;\sin\left(\omega_{0}t\right),\;\;\;\;j=1,2,$$

where $F_{jc}$ and $F_{js}$ are slowly varying cosine and sine components, respectively [37].

Substituting Eqs. (B-7) and (B-8) into Eqs. (B-5) and (B-6) and neglecting the second-order derivatives and higher-order frequency terms, we write the equations for the quadrature components of the motion of nanoparticles as

(B-9)
$${\dot{Q}}_{1}=-\gamma_{g}Q_{1}+sP_{1}-sP_{2}-\frac{F_{s1}}{2},$$

(B-10)
$${\dot{P}}_{1}=-\gamma_{g}P_{1}-sQ_{1}+sQ_{2}+\frac{F_{c1}}{2},$$

(B-11)
$${\dot{Q}}_{2}=-\left(\gamma_{g}-r\gamma_{g}-\gamma_{a}+6\gamma_{f}\left[Q_{2}^{2}+P_{2}^{2}\right]\right)Q_{2}-\frac{F_{s2}}{2},$$

(B-12)
$${\dot{P}}_{2}=-\left(\gamma_{g}+r\gamma_{g}-\gamma_{a}+6\gamma_{f}\left[Q_{2}^{2}+P_{2}^{2}\right]\right)P_{2}+\frac{F_{c2}}{2},$$

where *r* = *fω*_0_/*γ*_*g*_ is the squeezing strength and *s* = *g*/*kd*_0_ is the coupling strength.

## Appendix C: Fokker–Planck equation

We make use of the unidirectionality condition *S*_2_ = 0 and proceed to transform the master equation, as in Eq. (A-1), into a Fokker–Planck (FP) equation.

At first, we make a unitary transformation $W={\text{e}}^{-i\sum_{j=1}^{2}\omega_{j}b_{j}^{†}b_{j}t}$ and transfer the master equation into the interaction picture. Then, we use the relation between the density matrix *ρ* and the corresponding probability distribution function P in terms of the Bargmann states [59], given by

(C-1)
$$\rho =\int d^{2}\alpha_{j}\|\alpha_{j}〉〈\alpha_{j}\|\;{\text{e}}^{-\alpha_{j}\alpha_{j}^{*}}P\left(\alpha_{j},\alpha_{j}^{*}\right).$$

Here, ‖*α*_*j*_⟩ represents a Bargmann state with amplitude *α*_*j*_ and employ the following properties representing how the action of the creation and annihilation operators on *ρ* is reflected in the space of variables of P :

(C-2)
$$\begin{array}{l} b_{j}\rho \;↔\;\alpha_{j}P\left(\alpha_{j},\alpha_{j}^{*}\right),\\ b_{j}^{†}\rho \;↔\;\left(\alpha_{j}^{*}-\frac{\partial }{\partial \alpha_{j}}\right)P\left(\alpha_{j},\alpha_{j}^{*}\right),\\ \rho b_{j}\;↔\;\left(\alpha_{j}-\frac{\partial }{\partial \alpha_{j}^{*}}\right)P\left(\alpha_{j},\alpha_{j}^{*}\right),\\ \rho b_{j}^{†}\;↔\;\alpha_{j}^{*}P\left(\alpha_{j},\alpha_{j}^{*}\right). \end{array}$$

$P\left(\alpha_{j},\alpha_{j}^{*}\right)$ is the probability distribution function with *α*_*j*_ = *Q*_*j*_ + *iP*_*j*_ and $\alpha_{j}^{*}$ being its complex conjugate [59]. Next, for simplicity, we assume *γ*_*g*1_ = *γ*_*g*2_ = *γ*_*g*_, *A*_*t*1_ = *A*_*t*2_ = *A*_*t*_ and make use of Eqs. (C-1) and (C-2) to write an approximate FP equation for the coupled system as

(C-3)
$$\begin{array}{ll} \frac{\partial P}{\partial t} & \approx \frac{A_{t1}}{4}\frac{\partial^{2}}{\partial Q_{1}^{2}}P+\gamma_{g}\frac{\partial }{\partial Q_{1}}Q_{1}P+\frac{A_{t1}}{4}\frac{\partial^{2}}{\partial P_{1}^{2}}P\\ & \;+\gamma_{g}\frac{\partial }{\partial P_{1}}P_{1}P-s\frac{\partial }{\partial Q_{1}}P_{1}P+s\frac{\partial }{\partial P_{1}}Q_{1}P\\ & \;-s\frac{\partial }{\partial P_{1}}Q_{2}P+s\frac{\partial }{\partial Q_{1}}P_{2}P+J_{s}\frac{\partial^{2}}{\partial Q_{1}\partial Q_{2}}P\\ & \;+J_{s}\frac{\partial^{2}}{\partial P_{1}\partial P_{2}}P+\frac{A_{t2}}{4}\frac{\partial^{2}}{\partial Q_{2}^{2}}P+\left(\gamma_{g}-\gamma_{g}r-\gamma_{a}\right)\frac{\partial }{\partial Q_{2}}Q_{2}P\\ & \;+\frac{A_{t3}}{4}\frac{\partial^{2}}{\partial P_{2}^{2}}P+\left(\gamma_{g}+\gamma_{g}r-\gamma_{a}\right)\frac{\partial }{\partial P_{2}}P_{2}P\\ & \;+6\gamma_{f}\frac{\partial }{\partial Q_{2}}Q_{2}\left(Q_{2}^{2}+P_{2}^{2}\right)P+6\gamma_{f}\frac{\partial }{\partial P_{2}}P_{2}\left(Q_{2}^{2}+P_{2}^{2}\right)P, \end{array}$$

where $P\equiv P\left(Q_{1},P_{1},Q_{2},P_{2}\right)$ , *A*_*t*1_ = *A*_*t*_ + *D*_*p*_ − *γ*_*g*_, *A*_*t*2_ = *A*_*t*_ + *D*_*p*_ − *γ*_*g*_ + *γ*_*a*_ + *γ*_*g*_ *r* + 2Γ_*a*_ + 12Γ_*f*_, *A*_*t*3_ = *A*_*t*_ + *D*_*p*_ − *γ*_*g*_ + *γ*_*a*_ − *γ*_*g*_ *r* + 2Γ_*a*_ + 12Γ_*f*_. Considering actual experimental values as in Refs. [10], [33], [42], we can safely assume *A*_*t*1_ ≈ *A*_*t*_, *A*_*t*2_ ≈ *A*_*t*_, and *A*_*t*3_ ≈ *A*_*t*_.

## Appendix D: Analysis for squeezed states

In the case of the generation of squeezed states, we consider *γ*_*a*_ = 0 and *γ*_*f*_ = 0 in the above FP equation, Eq. (C-3), and find an analytical solution for it in the steady-state limit. By assuming *A*_*t*_ ≫ (*γ*_*g*_, *s*, *J*_*s*_) and *s* > *γ*_*g*_ and then making use of the drift-diffusion approach for the interacting system as in Ref. [60], we find such solutions for the probability distribution functions P of both nanoparticles as

(D-1)
$$P_{2}\approx C_{2}\exp\left[-\frac{2}{A_{t}}\left\{\left(\gamma_{g}-\gamma_{g}r\right)Q_{2}^{2}+\left(\gamma_{g}+\gamma_{g}r\right)P_{2}^{2}\right\}\right]$$

(D-2)
$$\begin{array}{ll} P_{1} & \approx C_{1}\exp\left[-\frac{2}{A_{t}}\left\{\left(\gamma_{g}-\gamma_{g}r+\frac{\gamma_{g}^{2}}{s^{2}}\left(\gamma_{g}+\gamma_{g}r\right)\right)Q_{1}^{2}\right\}\right]\\ & \;\times \exp\left[-\frac{2}{A_{t}}\left\{\left(\gamma_{g}+\gamma_{g}r+\frac{\gamma_{g}^{2}}{s^{2}}\left(\gamma_{g}-\gamma_{g}r\right)\right)P_{1}^{2}\right\}\right]\\ & \;\times \exp\left[-\frac{2}{A_{t}}\frac{4\gamma_{g}^{2}r}{s}Q_{1}P_{1}\right], \end{array}$$

where $P_{2}\equiv P_{2}\left(Q_{2},P_{2}\right)$ and $P_{1}\equiv P_{1}\left(Q_{1},P_{1}\right)$ are the reduced probability distribution function for nanoparticle 2 and nanoparticle 1, respectively [60]. Further, *C*_1_ and *C*_2_ are the normalization constants. The corresponding plots are shown in Figure D-6.

To quantify the squeezing in the coupled levitated system, we calculate mean values as

(D-3)
$$\left\langle O\right\rangle =\frac{\int O\;P_{j}\left(O\right)\;dO}{\int P_{j}\left(O\right)\;dO},\;\;\;\;O\in \left\{Q_{2},P_{2},Q_{1},P_{1}\right\},$$

and derive analytical expressions for the variances of the quadrature components of both the nanoparticles, leading to

(D-4)
$$\sigma_{Q_{2}}^{2}=〈Q_{2}^{2}〉-〈Q_{2}{〉}^{2}=\frac{A_{t}}{4\gamma_{g}\left(1-r\right)},$$

(D-5)
$$\sigma_{P_{2}}^{2}=〈P_{2}^{2}〉-〈P_{2}{〉}^{2}=\frac{A_{t}}{4\gamma_{g}\left(1+r\right)},$$

(D-6)
$$\begin{array}{ll} \sigma_{Q_{1}}^{2} & =〈Q_{1}^{2}〉-〈Q_{1}{〉}^{2}\\ & =\frac{A_{t}s^{2}}{4\gamma_{g}^{2}\left(1-r^{2}\right){\left(\gamma_{g}^{2}+s^{2}\right)}^{2}}\left[\gamma_{g}^{3}\left(1-r\right)+\gamma_{g}\left(1+r\right)s^{2}\right]\\ & \approx \frac{A_{t}}{4\gamma_{g}\left(1-r\right)}\;\;\;\;\text{for}\;\;s\gg \gamma_{g}, \end{array}$$

(D-7)
$$\begin{array}{ll} \sigma_{P_{1}}^{2} & =〈P_{1}^{2}〉-〈P_{1}{〉}^{2}\\ & =\frac{A_{t}s^{2}}{4\gamma_{g}^{2}\left(1-r^{2}\right){\left(\gamma_{g}^{2}+s^{2}\right)}^{2}}\left[\gamma_{g}^{3}\left(1+r\right)+\gamma_{g}\left(1-r\right)s^{2}\right]\\ & \approx \frac{A_{t}}{4\gamma_{g}\left(1+r\right)}\;\;\;\;\text{for}\;\;s\gg \gamma_{g}. \end{array}$$

Finally, to measure how close the transported state of nanoparticle 1 is to the original state of nanoparticle 2, we evaluate the corresponding fidelity as [61], [62]

(D-8)
$$F=\frac{\iint P_{1}\left(Q,P\right)\;P_{2}\left(Q,P\right)\;dQ\;dP}{\iint {\left[P_{2}\left(Q,P\right)\right]}^{2}\;dQ\;dP}.$$

## Appendix E: Analysis for random-phase coherent states

For creating and transporting random-phase coherent states, we consider *r* = 0 in Eq. (C-3) and use the method as suggested in the above section along with the assumption that *A*_*t*_ ≫ (*γ*_*g*_, *γ*_*a*_, *s*, *J*_*s*_) and *s* > *γ*_*g*_ and derive the analytical solution for the probability distribution function P of both nanoparticles in the steady-state limit. This gives

(E-1)
$$\begin{array}{ll} P_{2} & \approx C_{4}\exp\left[-\frac{2}{A_{t}}\left\{\left(\gamma_{g}-\gamma_{a}\right)\left(Q_{2}^{2}+P_{2}^{2}\right)\right\}\right]\\ & \;\times \exp\left[-\frac{2}{A_{t}}\left\{3\gamma_{f}{\left(Q_{2}^{2}+P_{2}^{2}\right)}^{2}\right\}\right], \end{array}$$

(E-2)
$$\begin{array}{ll} P_{1} & \approx C_{3}\exp\left[-\frac{2}{A_{t}}\left\{\left(\gamma_{g}-\gamma_{a}\right)\left(1+\frac{\gamma_{g}^{2}}{s^{2}}\right)\left(Q_{1}^{2}+P_{1}^{2}\right)\right\}\right]\\ & \;\times \exp\left[-\frac{2}{A_{t}}\left\{3\gamma_{f}{\left(1+\frac{\gamma_{g}^{2}}{s^{2}}\right)}^{2}{\left(Q_{1}^{2}+P_{1}^{2}\right)}^{2}\right\}\right]. \end{array}$$

Here, $P_{2}\equiv P_{2}\left(Q_{2},P_{2}\right)$ and $P_{1}\equiv P_{1}\left(Q_{1},P_{1}\right)$ are the reduced probability distribution functions for the nanoparticle 2 and nanoparticle 1, respectively [60], and *C*_3_ and *C*_4_ are the normalization constants. The corresponding plots are shown in Figure E-7. By using Eq. (D-3), we can find the steady-state phonon populations for both nanoparticles as

(E-3)
$$N_{2}=〈Q_{2}^{2}+P_{2}^{2}〉=\frac{\gamma_{a}-\gamma_{g}}{6\gamma_{f}},$$

(E-4)
$$\begin{array}{ll} N_{1} & =〈Q_{1}^{2}+P_{1}^{2}〉=\frac{\left(\gamma_{a}-\gamma_{g}\right)s^{2}}{6\gamma_{f}\left(\gamma_{g}^{2}+s^{2}\right)}\\ & \approx \frac{\gamma_{a}-\gamma_{g}}{6\gamma_{f}}\;\;\;\;\text{for}\;\;s\gg \gamma_{g}, \end{array}$$

where *N*_2_ (*N*_1_) represents the steady-state phonon number for nanoparticle 2 [1].
